# Impact of ENT complications on COVID-19 patients in a tertiary care center, Coimbatore: a retrospective cross-sectional study

**DOI:** 10.1186/s43163-023-00458-4

**Published:** 2023-06-12

**Authors:** K. Karthikeyan, Kishore Kumar P., V. Ravisankar, A. Dayanand, Jeril Lasington, Jagannath Soundararajan

**Affiliations:** PSG Institute of Medical Sciences and Research, Peelamedu, Coimbatore, Tamil Nadu 641004 India

**Keywords:** COVID-19, Mucormycosis, Diabetes, ENT complications

## Abstract

**Background:**

Infection by severe acute respiratory syndrome coronavirus 2 (SARS-CoV-2) presents with respiratory tract-related symptoms such as fever, cough, dyspnea, and chest tightness that could progress rapidly to acute respiratory distress syndrome (ARDS). However, coronavirus disease 2019 (COVID-19) also causes several upper respiratory tract-related symptoms including nasal congestion, sore throat, and smell dysfunction. Incidence of mucormycosis in COVID-19 patients was also reported during the second wave along with other otorhinolaryngology-related complications.

**Aim:**

This study aimed to determine different otorhinolaryngology-related complications reported in COVID-19 cases in a tertiary care center and estimation of incidence, age, gender distribution, risk factors, treatment, follow-up, and mortality status of the study participants.

**Methodology:**

Patients who recovered from COVID-19 disease and developed complications such as long-lasting anosmia, septal abscess, epistaxis, rhino nasal mucormycosis, and voice changes were selected for the study. After obtaining patient medical records, the recorded history, examination, and investigations done related to incidence, associated risk factors, age, and gender distribution of ENT (Ears, Nose, Throat) complications were collected, and this data was analyzed.

**Results:**

The present study involved 100 patients between 18 and 65 years of age which included 78% males and 22% females. Of these participants, 92% reported mucormycosis as the most common complication, with diabetes being the risk factor in majority of the participants. During the course of the study, 10% participants had passed away due to complications.

**Conclusion:**

Early identification of highly sensitive post-COVID-19 ENT complications is essential to reduce mortality and morbidity among vulnerable populations.

## Background

Severe acute respiratory syndrome corona virus 2 (SARS-CoV-2) originated in Wuhan, China as an outbreak of acute community-acquired atypical pneumonia towards the end of 2019 and was linked with a seafood market situated there. By January 2020, SARS-CoV-2 was reported in China, across East and Southeast Asia, Europe, and the USA [1]. The primary mode of transmission was identified to be direct contact and via droplets of respiratory mucus secretion with symptoms occurring after an incubation period of 2–14 days. In extreme cases, period from onset of symptoms to death of individual was between 6 and 41 days depending on the age and status of the immune system [2].

According to recent evidence, long COVID, or post-COVID syndrome was reported in patients who recovered from COVID but continued to present signs and symptoms of illness 4 weeks after the initial diagnosis of SARSCoV-2 infection, which could not be explained by other causes. This condition had a prevalence of around 10–30% in patients with a recent history of SARS-CoV-2 infection and presented with pulmonary, hematologic, cardiovascular, neuropsychiatric, renal, endocrine, gastrointestinal, hepatobiliary, and dermatologic involvement as well as chronic multisystem inflammatory syndrome in children (MIS-C) and these signs and symptoms lasted for several months [3].

Coronavirus disease 2019 (COVID-19) presented mainly with lower respiratory tract-related symptoms such as fever, cough, dyspnea, and chest tightness that could progress rapidly to acute respiratory distress syndrome (ARDS) [4]. The inner ear portion has been reported to be vulnerable to viruses; recent data linked sensorineural hearing loss (SNHL), tinnitus, and/or vertigo, with SARS-CoV-2 infection in patients. ENT (Ears, Nose, Throat)-related symptoms associated during and following COVID-19 infection include sensorineural hearing loss (SNHL), tinnitus, and/or vertigo [5].

The emergence of COVID-19 has also coincided with reports of fungal infections such as aspergillosis and mucormycosis, especially among critically ill patients treated with steroids [6]. It has also coincided with the increased incidence of acute invasive fungal rhinosinusitis (AIFRS) which is an aggressive infection associated with high mortality rate [7]. New symptomatic manifestations and complications of the disease continue to be identified and described in medical literature. This study aimed to characterize the different long lasting ENT-related complications in COVID-19 recovered patients admitted at a tertiary care hospital in Coimbatore, Tamil Nadu, India and estimate the incidence, age and gender distribution, risk factors, treatment, follow-up, and mortality status of study participants. Early identification of these highly sensitive conditions is key to allow for optimal treatment and improved outcomes.

## Methods

A retrospective descriptive study was conducted between August 2020 and June 2021 in the Department of Otorhinolaryngology, COVID Review Clinic, at a tertiary healthcare center. The study involved COVID-19-positive patients with ENT complications or long-lasting ENT symptoms which lasted more than 28 days after the onset of COVID-19 infection.

Sample size was calculated for the study using the prevalence reported in a study conducted by Varia et al. [8] The previously study conducted by Varia et al. reported that 73% participants had loss of smell and taste, and using this prevalence rate, the sample size was calculated using the formula:

$$n=4\times\mathrm p\times\mathrm q\,/\,\mathrm{d}^{2}\;\mathrm{with}\;p=73,\;\mathrm q=100-p=100-73=27,\mathrm d=\mathrm{relative}\;\mathrm{precision},\mathrm i.\mathrm e.,15\%\;\mathrm o\mathrm f\;p=15\times73/100=10.95$$

$$n=4\times 73\times 27/10.95\times 10.95=7884/119.9=65$$

with a 20 addition to make up for non–respondents: 65 × 20/100 = 13

So the required sample size was 65 + 13 = 88. However, a sample size of 100 patients was considered for the present study.

The inclusion criteria for the present study included COVID-19-positive patients with ENT complications between 18 and 65 years of age. Infants, toddlers, and adolescents up to the age of 18 years and adults above 65 years were excluded from the study.

**Fig. 1:**
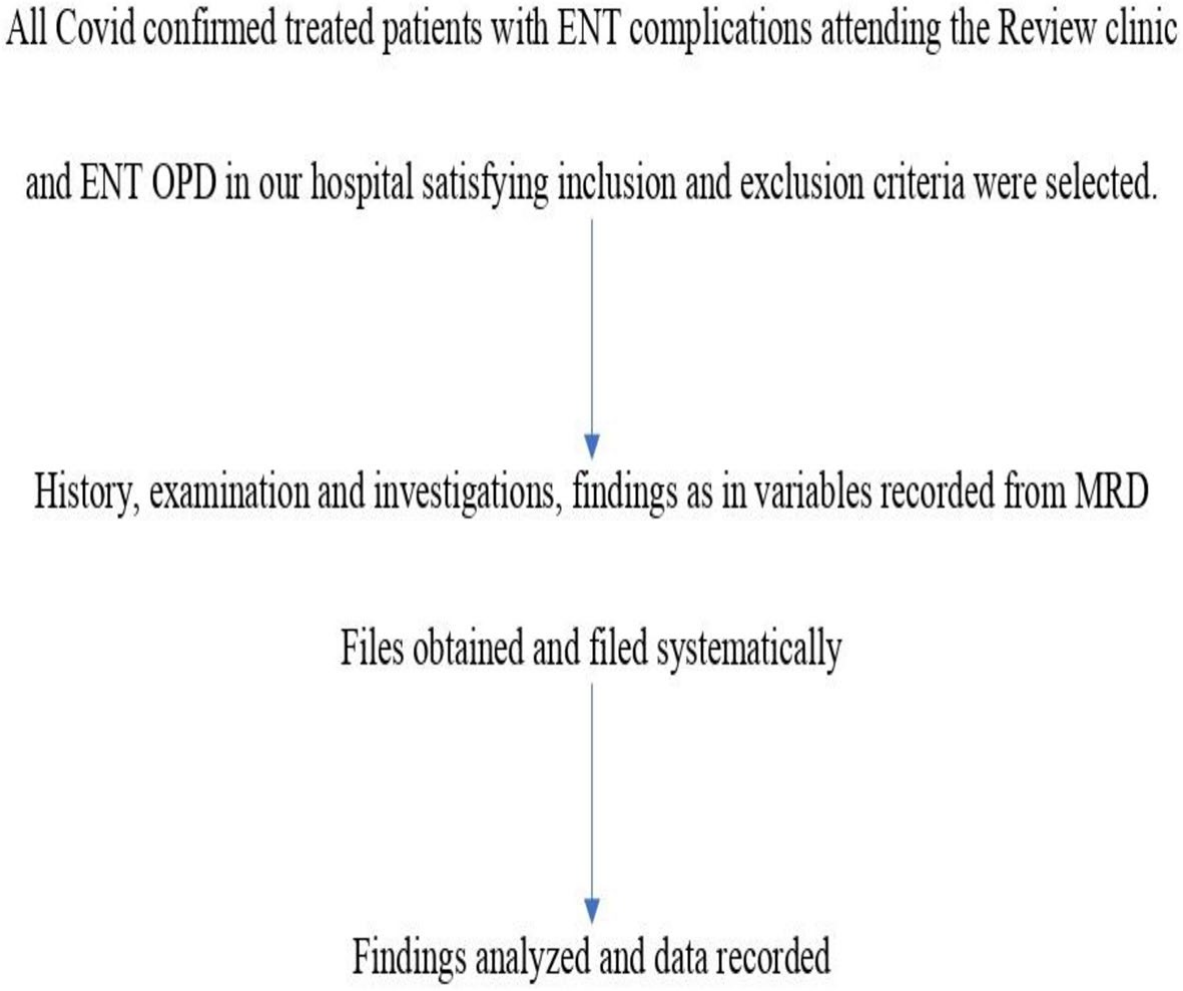
Flowchart of the study

After obtaining ethical committee approval (Ref no: PSG/IHEC/2021/Appr/Exp/049, Project no: 21/056), 100 patients who had developed spontaneous ENT complications like long lasting anosmia (COVID-19-related olfactory dysfunction lasting more than 30 days), septal abscess, epistaxis, rhino nasal mucormycosis, or voice changes were selected for the study. Mucormycosis was diagnosed on the basis of clinical signs and symptoms, radiological signs (magnetic resonance imaging [MRI] and CT scans), and biopsy of the lesion by histopathological confirmation. Selected participants did not show the presence of any other complications. Patient information was procured via files obtained from the medical record department (MRD), and these were filed systematically. Variables recorded as part of the study included name, age, gender, occupation, complaints and duration of illness, past history, personal history, family history, general examination, complete head and neck examination, ENT examination, cranial nerve examination, imaging (MRI or high-resolution computer tomography [HRCT] if available), complete blood counts, ESR, and extended diabetic profile in diabetic patients. The flowchart of the present study is depicted in Fig. 1.

### Statistical analysis

Data was analyzed using IBM SPSS Statistics for Windows, Version 23.0. (Armonk, NY: IBM Corp). To describe the data, frequency and percentage analysis were used for categorical variables and the mean and standard deviation were used for continuous variables.

## Results

In the present study which included 100 COVID-19-positive patients, majority of the study participants (78%) were males with the mean age of all study participants being 52 ± 12 years. Based on the age of selected participants who presented with complications, 34% cases belonged to the age group of 41–50 years while 28% belonged to the age group of 51–60 years. Among all selected cases, type 2 diabetes mellitus was the predominant risk factor as it was present in 93% of cases while the remaining 7% reported no associated risk factors.

Of the 100 cases with complications, 92% cases suffered from mucormycosis, septal abscess was seen in 3% participants, unilateral vocal cord palsy was seen in 2% participants, long lasting anosmia was seen in 2% of the participants, and epistaxis was seen in 1% participants.

All the 92% mucormycosis cases underwent treatment in the form of endoscopic nasal debridement. Incision and drainage were performed to treat all the 3% septal abscess cases, and the remaining 5% cases who presented with long lasting anosmia, epistaxis, and unilateral vocal cord palsy were managed conservatively. Among the participants, about 10% cases passed away mainly due to post COVID-19 complications like respiratory failure or pneumonia. Post-treatment, nearly 90% patients were regular for follow-up (Table 1).

**Table 1:** Table depicting data on patients with ENT complications

	**Frequency**	**Percent**
**Age distribution of study participants**		
Up to 40 years	15	15.0
41–50 years	34	34.0
51–60 years	28	28.0
61–70 years	16	16.0
71–80 years	7	7.0
Total	100	100.0
**Gender distribution of study participants**		
Female	22	22.0
Male	78	78.0
Total	100	100.0
**Distribution of ENT complications**		
Anosmia	2	2.0
Epistaxis	1	1.0
Mucormycosis	92	92.0
Septal abscess	3	3.0
Vocal cord palsy (unilateral-right)	2	2.0
Total	100	100.0
**Risk factors associated with ENT complications**		
Nil	7	7.0
Type 2 diabetes mellitus	93	93.0
Total	100	100.0
**Treatment given**		
Conservative	5	5.0
Endoscopic nasal debridement	92	92.0
Incision and drainage	3	3.0
Total	100	100.0
**Mortality**		
Alive	90	90.0
Passed away	10	10.0
Total	100	100.0
**Follow-up**		
Lost follow-up	10	10.0
In follow-up	90	90.0
Total	100	100.0

With respect to mucormycosis patients, the mean age of the study participants was 53 ± 12 years. Among patients who presented with mucormycosis, 32.6% study participants belonged to the age group of 41–50 years and 27.2% belonged to the age group of 51–60 years. Among the participants who presented with mucormycosis, 78.3% were males. Type 2 diabetes mellitus was the predominant risk factor and was present in 98.9% cases, while the remaining 1.1% had no associated risk factors. Post-treatment, 10.9% cases passed away mainly due to post-COVID-19 complications like respiratory failure and pneumonia and 89.1% patients were under regular follow-up (Table 2).

**Table 2:** Table depicting data on patients with mucormycosis

	**Frequency**	**Percent**
**Age distribution of mucormycosis patients**		
Up to 40 years	14	15.2
41–50 years	30	32.6
51–60 years	25	27.2
61–70 years	16	17.4
71–80 years	7	7.6
Total	92	100.0
**Gender distribution of mucormycosis cases**		
Female	20	21.7
Male	72	78.3
Total	92	100.0
**Risk factor associated with mucormycosis**		
Nil	1	1.1
Type 2 diabetes mellitus	91	98.9
Total	92	100.0
**Mortality in mucormycosis cases**		
Alive	82	89.1
Passed away	10	10.9
Total	92	100.0
**Follow up in mucormycosis cases**		
Lost follow-up	10	10.9
In follow-up	82	89.1
Total	92	100.0

## Discussion

Infection by the COVID-19 virus results in a clinically wide range of manifestations ranging from no symptoms to septic shock and multi-organ dysfunction. The nasal, nasopharyngeal, and/or the oropharyngeal tissues are the main harbor sites of infection, main sites for taking sample for testing, and main sources of transmission of infection [4]. One of the main signs of COVID-19 infection was shown to be anosmia, with average onset seen 3 months after initial infection. A previous study has shown that a window of 1–2 months after onset of symptoms showed a substantial improvement in the olfactory abilities of patients after recovery from COVID-19 infection [9]. Another study showed a marked clinical improvement in post-COVID-19 infection anosmia patients by means of using 0.9% sodium gluconate as an intranasal spray [10]. A study conducted in Italy reported that close to 55% patients showed 3 or more post-COVID symptoms within 60 days following early COVID-19 onset [11]. Any COVID-19 related olfactory dysfunction lasing more than 30 days was considered as long last anosmia in the present study.

The present study identified 100 COVID-19 patients who developed spontaneous ENT complications like long lasting anosmia, septal abscess, epistaxis, rhino-nasal mucormycosis, and voice change. The overall mean age of the study participants with post-COVID-19 ENT complications was 52 ± 12 years. This is in agreement with a study conducted by Bhanuprasad et al. [12] wherein among 132 post-COVID-19 participants, the mean age of participants who presented with ENT complications was 50.52 ± 11.66 years. In studies on post-COVID-19 vocal cord palsy by Tin et al. [13] epistaxis by Dell’Era et al*.* [14], and anosmia by Vaira et al. [15] the mean age of the participants was 58 years, 71.5 years, and 51.2 years, respectively. These results add to the already established evidence that COVID-19 incidence increases with advanced age and ultimately results in increased complications due to comorbidities related with advanced age. The patients that reported to the COVID Review Clinic where the study took place all fell within 65 years of age; hence, older patients could not be assessed as part of the study.

In the present study consisting of patients who presented with ENT complications, 78% were males and 22% females. This is in agreement with a study conducted by Bhanuprasad et al. [12] which included 132 participants presenting with mucormycosis consisting of 78% males. This was also similar to a study involving patients with epistaxis by Dell’Era et al*.* [14] which included 77% males. A systematic review conducted by Pal et al*.* also showed that COVID-19-associated mucormycosis (CAM) cases were more prominent among males (78%) [16]. COVID-19 incidence has been found to be more common in males, possibly due to differences mediated by various factors like sex hormones and increased expression of coronavirus receptors (ACE 2). Additionally, life style differences like higher levels of smoking and drinking among men as compared to women could be a contributing factor. As COVID-19 incidence is more in male population, related complications could also present with higher incidence as shown in the present study [17].

In the present study, only 1% participants showed development of epistaxis. This is in contrast with the results from a study by Dell’Era V et al*.* where about 30% of the COVID positive patients presented with spontaneous epistaxis. The use of oxygen therapy in the treatment of COVID-19 could be considered as the predominant cause for epistaxis incidence [14]. Epistaxis has also been reported as a common adverse event post-vaccine administration in some individuals [18].

Septal abscess incidence was seen in a small proportion of COVID-19 patients. The etiology of septal abscess formation is majorly credited to trauma which becomes infected, followed by causes secondary to sinusitis, vestibulitis, and dental pathologies, with spontaneous incidence being a rare occurrence. The incidence of septal abscess during the pandemic could be credited to trauma which was not addressed due to stigma toward hospital visits [19]. Long lasting anosmia was also reported in a small number of participants; its occurrence among COVID-19 patients is considered as one of the telltale signs of infection and hence is expected to occur as a complication of COVID-19 infection [9]. The incidence reported in the present study is much lower when compared to the study conducted by Klopfensein et al*.* that showed anosmia incidence of 47% with 98% participants reported to have recovered within 28 days [20]. The results of the present study are also in contrast with those of multiple studies included in a review where the incidence of anosmia ranged between 33.9% and 68% [21]. A study on the recovery of olfactory dysfunction showed that 24.1% participants did not recover olfaction after 60 days of onset and at 6 months and 4.7% patients had not recovered their sense of smell. The incidence seen at 6-month time period is close to that reported in the present study [22]. This could also be categorized as very long-lasting anosmia. Another study showed that anosmia was observed as a post-COVID symptom in 12.4% participants 4 months after the onset of COVID-19 infection [23]. This sort of wide discrepancy in the recovery rate of COVID-19 patients with respect to anosmia symptoms is an avenue of research that should be further studied.

The incidence of vocal cord paralysis was reported in a minority of participants. A sparse incidence of vocal cord paralysis has been reported in case series conducted by Zamzam et al*.* and Rapoport et al*.* which suggested that viral infections like COVID-19 could result in post-viral vagal neuropathy and form the etiology of vocal cord paralysis [24, 25].

Mucormycosis was diagnosed as the predominant post-COVID-19 ENT complication accounting for more than 90% of all cases. All mucormycosis diagnosis were confirmed by histopathological examination of associated lesions. This result is in agreement with the results of a study conducted by Muthu et al*.* [26] in which among 233 post-COVID-19 patients, rhino-orbital mucormycosis was the most frequent presentation and accounted for 89% of cases. This is also in agreement with a study conducted by Gupta which reported that all mucormycosis patients involved in the study had preceding COVID infection and 25.7% were diagnosed during the course of their COVID treatment [27]. According to a review by Islam et al., 14,872 instances of CAM were reported from India which makes up for a majority (71%) of all reported cases [28]. The results of the present study help reinforce evidence where mucormycosis was found to be the most common ENT-related complication among post-COVID-19 patients.

More than 90% patients suffered from diabetes mellitus as an underlying risk factor which was higher than the proportion found in a multicentric COVID-mucormycosis study by Patel et al*.* [29] conducted in India during the pandemic. Other studies on post-COVID-19 vocal cord palsy by Tin et al. and Vaira et al*.* [13, 15] showed majority patients suffering from type 2 diabetes as an underlying risk factor. A previously conducted systematic review also agrees with the results of the present study by reporting that 85% of the patients suffering from CAM reported diabetes mellitus as the major risk factor for mucormycosis [16]. Another study by Gupta also reported that 71.6% of the included mucormycosis patients were diabetic which is in agreement with the results of the present study and adds to the existing evidence of diabetes being a risk factor for developing mucormycosis [27]. According to the results of a meta-analysis, diabetes mellitus was found to be a major predisposing factor for mucormycosis occurrence with 70% patients with rhino-orbital–cerebral mucormycosis suffering from diabetes [30]. The participants who presented to the ENT department in the given time period of the study consisted mainly of diabetic patients. The participants with other comorbidities could have been missed out due of them being redirected to respective speciality departments for treatment; this could explain the high incidence of diabetics in the present study.

With regard to treatment modalities, all mucormycosis patients underwent endoscopic nasal debridement, while all the patients with septal abscesses underwent incision and drainage. The patients with long lasting anosmia, epistaxis, and vocal cord palsy were managed by conservative methods.

In a study conducted by Muthu et al. [24] on post-COVID mucormycosis patients, surgical treatment was conducted on about 72.1% of the study participants which is in agreement with the present study as it consisted of a huge majority of involved patients. Another study on COVID-positive septal abscess cases by Maan et al. [31] showed that the cases were managed by incision and drainage which is similar to the results of the present study. Studies on post-COVID vocal cord palsy by Tin et al. [13] epistaxis by Dell’Era et al. [14], and anosmia by Vaira et al. [15] reported that patients were managed conservatively which is also in complete agreement with the results of the present study.

The results of the present study showed that 90% of cases were regularly followed up, whereas the remaining mucormycosis cases had passed away mainly due to post-COVID complications. This was similar to the results of a study conducted by Garg et al. with respect to COVID-19-associated mucormycosis which reported an overall mortality of 16.3% [32]. Another study showed that 12.1% of included mucormycosis patients had passed away due to disease/underlying medical and treatment-related complications and this finding is in agreement with the results of the present study [27]. A systematic review reported CAM mortality to be 34% which is much higher than that reported in the present study [14]. These differences in mortality rate could be due to factors like differences in medical facilities among hospitals or increased burden on hospitals.

A previously systematic review concluded that 72% of all CAM cases were from India (highest global burden) which could be explained by the high incidence of diabetes, with India being home to about 77 million people suffering with diabetes with a nationwide prevalence of 7.3% [16]. This information could help in assessing the extent and prognosis of the post-COVID complications which can help in reducing associated mortality and morbidity.

The strength of the present study was that the study duration of 1 year facilitated collection of vast amounts of data as it coincided with both first and second waves of COVID-19; the second wave showing a rise in cases of mucormycosis in COVID-19 patients which was one of the major findings. Any form of recall bias was avoided with the use of hospital records as the primary source of data. The limitation of the study included selection bias caused by only patients visiting the ENT department of one COVID review clinic being included in the study and the small sample size, the age criteria implemented did not include patients below 18 years of age even though children and younger adults were found to be highly susceptible to contract COVID-19 and also, the retrospective nature of the study and missing radiological findings from some patients contributes to the study limitations, a prospective study would provide more strength to a study following up associated complications. The clinical implications of the study are that the results can be used to ascertain the different ENT-related complications among adults after recovering from COVID-19, and this may help in developing necessary aftercare protocols to ensure reduced incidence of these complications, especially in patients suffering from diabetes which was found to be a high-risk factor for mucormycosis. Studies involving more COVID clinics and with longer study duration can help in providing more data on the incidence and effects of these complications.

## Conclusion

COVID-19 has affected a huge portion of individuals worldwide and the long-term clinical effects of the disease even after complete recovery are mainly unknown. In the present study, mucormycosis was the predominant complication with the main risk factor being type 2 diabetes mellitus, with endoscopic nasal debridement being the most common treatment modality. This evidence can help in ensuring more thorough follow up for patients with diabetes as a comorbidity. Hence, the present study concludes that early identification of these highly sensitive post-COVID ENT complications is the key for optimal treatment and improved outcome, to avoid mortality and morbidity among vulnerable populations.

## Data Availability

The datasets used and/or analyzed during the current study are available from the corresponding author on reasonable request.
